# Different Cardiac Anomalies in Mother and Son with 4q-Syndrome

**DOI:** 10.1155/2015/932651

**Published:** 2015-08-31

**Authors:** Marcello Marcì, Angela Guarina, M. Cristina Castiglione, Nicola Sanfilippo

**Affiliations:** ^1^Cardiology Department, Ospedali Riuniti Villa Sofia Cervello, 90100 Palermo, Italy; ^2^Pediatric Clinic, Policlinico Universitario, Palermo, Italy

## Abstract

We report a female patient with asymptomatic cor triatriatum sinister, associated with 4q34.3 deletion. Her child, carrying the same imbalance, suffers from tetralogy of Fallot. To the best of our knowledge, this is the first reported case of cor triatriatum associated with deletion of the long arm of the chromosome 4; furthermore, the majority of patients with chromosome 4 long arm syndrome have de novo deletions and only few familial cases have been reported so far.

## 1. Introduction

Terminal deletion of chromosome 4q is a rare condition with an estimated incidence of 1 : 100,000 [1]. A wide range of phenotypic manifestations has been reported from anomalies of skull and limbs to genitourinary malformations; mental and growth retardation are almost invariably associated [1–3].

Moreover, a high frequency of congenital heart disease (CHD) has been demonstrated in patients with 4q-syndrome, particularly in those with deletions encompassing the region 4q33.

We present two patients, a mother and her son, carrying the same 4q34.3 deletion but with different phenotypes.

The child suffers from tetralogy of Fallot, while the mother has nonobstructive cor triatriatum sinister. This latter is one of the rarest congenital cardiac malformations, in which the left atrium is divided by a thin membrane into two chambers communicating through one or more openings.

## 2. Case Report

Our institution follows a 1-year-old male child with tetralogy of Fallot, born to a 35-year-old woman after an uncomplicated pregnancy. He is the only child of unrelated parents. The family history was negative for CHD. Furthermore he had mild developmental delay and soft dysmorphic features, consisting in hypertelorism, low set ears, broad nasal bridge, and receding chin, while limbs and fingers were normal. Cytogenetic analysis of the proband and his parents was performed on cultured lymphocytes, using a validated oligonucleotide array comparative genomic hybridization Human Genome CGH 180 K Oligo Microarray kit (AMADID 22060, Agilent Technology) with a mean 13 Kb resolution.

**Figure 1:**
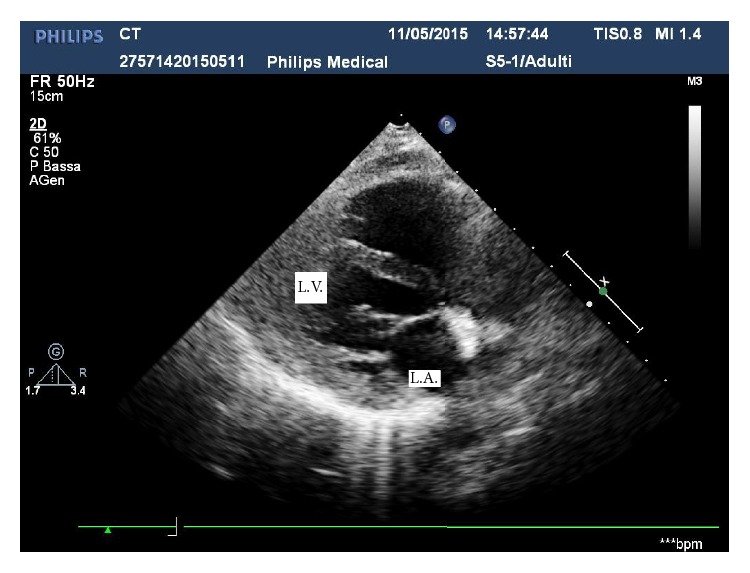
Long axis view. L.A.: left atrium; L.V.: left ventricle.

**Figure 2:**
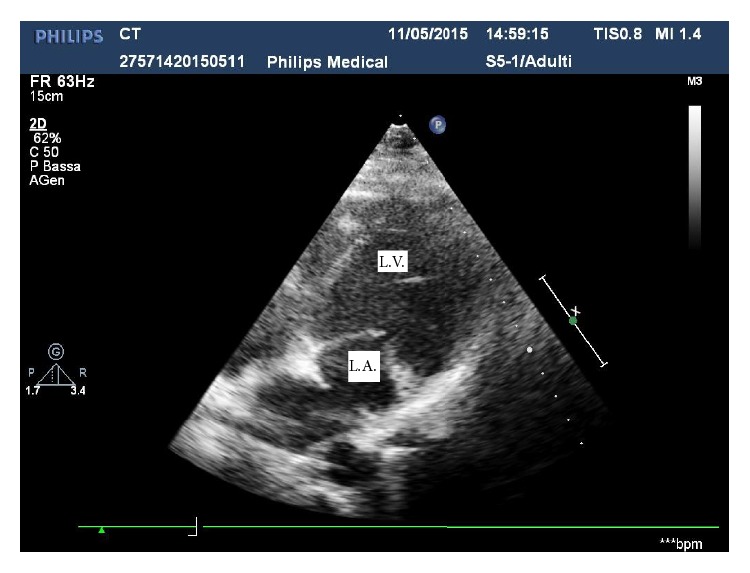
Four-chamber view. Inside the left atrium there is hyperechogenic structure. L.A.: left atrium; L.V.: left ventricle.

A male karyotype with a terminal 118 Kb deletion in the long arm of chromosome 4 (46,XY,del4q34.3) was demonstrated in the proband. Paternal chromosomes were normal. On the other hand his phenotypically and intellectually normal mother was found to carry the same imbalance. She underwent a cardiac examination in order to rule out a subclinical congenital malformation. Cardiac auscultation revealed a systolic murmur in the mitral area. The ECG was normal. Transthoracic echocardiography showed a thin perforated membrane dividing the left atrium into two chambers (Figures 1 and 2). Color-Doppler examination showed a mild mitral insufficiency and a nonturbulent flow through the diaphragm, while the pulsed-wave Doppler ruled out a significant obstruction between the two atrial chambers. Diagnosis of nonobstructive cor triatriatum sinister was established. Since the patient was asymptomatic surgical treatment was deemed unnecessary and only periodical examinations were recommended.

## 3. Discussion

We report a boy with tetralogy of Fallot in whom del4q34.3 was evidenced and the same imbalance was detected in his mother that has a nonobstructive cor triatriatum sinister.

Deletion of chromosome 4 long arm is a rare genetic aberration that determines the “chromosome 4q-syndrome,” characterized by some common phenotypic features consisting of mental retardation, dysmorphic facial anomalies, cleft palate, limb malformations, genitourinary, and gastrointestinal and cardiac anomalies [1–4].

Usually deletions proximal to 4q31 determine more severe malformations that often lead to miscarriage. On the other hand, patients with more distal deletions (4q33-4q34) are less severely affected, with minor abnormalities and mild mental retardation [1, 2].

Very often patients with 4q-syndrome have cardiac malformations; particularly defects of right ventricular outflow tract have been reported in 35% of these patients [1, 2, 4, 5].

The highest incidence of CHD, including pulmonary atresia, tetralogy of Fallot, ventricular septal defect, and aortic coarctation, has been found in patients with 4q33 deletion [5–7].

It has been postulated that congenital cardiac anomalies, mainly right outflow tract obstructions, are associated with deletions or duplications of gene of Heart and Neural crest Derivatives-expressed 2 (HAND2), mapped to 4q33 [5–9].

We describe two different cardiac malformations associated with del-4q34.3, namely, tetralogy of Fallot in a child and cor triatriatum in his mother. These cases present some interesting aspects; firstly in patients with 4q34 deletions CHD are quite uncommon [10–12] and in particular cor triatriatum has not been previously reported. Moreover, our patients demonstrate the considerable intrafamilial variability of the phenotypes determined by deletions of chromosome 4 long arm.

Tetralogy of Fallot is part of the spectrum of right ventricle obstruction; on the contrary cor triatriatum sinister is a left heart obstruction.

Cor triatriatum is very uncommon, representing only 0.1% of congenital cardiac malformation. In cor triatriatum the left atrium (cor triatriatum sinister) or the right atrium (cor triatriatum dexter) is divided into two chambers by a fibromuscular membrane. In cor triatriatum sinister, which is more common than dexter, the proximal atrial chamber receives the pulmonary veins, while the distal chamber communicates with the ventricle through the mitral valve.

The thin membrane, subdividing the atrium, usually has one or more openings.

When left ventricular filling is hindered by the diaphragm the anomaly determines symptoms of heart failure since infancy; otherwise some patients with nonobstructive lesions do not experience symptoms and disease can be diagnosed incidentally.

Further studies will be required to better characterize the cardiac anomalies associated with this 4q-syndrome.

## References

[1] Strehle E.-M., Bantock H. M. (2003). The phenotype of patients with 4q-syndrome. *Genetic Counseling*.

[2] Strehle E.-M., Gruszfeld D., Schenk D., Mehta S. G., Simonic I., Huang T. (2012). The spectrum of 4q-syndrome illustrated by a case series. *Gene*.

[3] Townes P. L., White M., Di Marzo S. (1979). 4q-Syndrome. *American Journal of Diseases of Children*.

[4] Lin A. E., Garver K. L., Diggans G. (1988). Interstitial and terminal deletions of the long arm of chromosome 4: further delineation of phenotypes. *American Journal of Medical Genetics*.

[5] Huang T., Lin A. E., Cox G. F. (2002). Cardiac phenotypes in chromosome 4q-syndrome with and without a deletion of the dHAND gene. *Genetics in Medicine*.

[6] Byatt S.-A., Baker E., Richards R. I., Roberts C., Smith A. (1997). Unbalanced t(4;11)(q32;q23) in a 34-year-old man with manifestations of distal monosomy 11q and trisomy 4q syndromes. *American Journal of Medical Genetics*.

[7] Borochowitz Z., Shalev S. A., Yehudai I., Bar-el H., Dar H., Tirosh E. (1997). Deletion (4)(q33*→*qter): a case report and review of the literature. *Journal of Child Neurology*.

[8] Shen L., Li X. F., Shen A. D. (2010). Transcription factor HAND2 mutations in sporadic Chinese patients with congenital heart disease. *Chinese Medical Journal*.

[9] Vincentz J. W., Barnes R. M., Firulli A. B. (2011). Hand factors as regulators of cardiac morphogenesis and implications for congenital heart defects. *Birth Defects Research A: Clinical and Molecular Teratology*.

[10] Yu C. W., Chen H., Baucum R. W., Hand A. M. (1981). Terminal deletion of the long arm of chromosome 4. Report of a case of 46, XY, del(4)(q31) and review of 4q-syndrome. *Annales de Genetique*.

[11] Tsai C.-H., Van Dyke D. L., Feldman G. L. (1999). Child with velocardiofacial syndrome and del (4)(q34.2): another critical region associated with a velocardiofacial syndrome-like phenotype. *American Journal of Medical Genetics*.

[12] Van Buggenhout G., Maas N. M. C., Fryns J.-P., Vermeesch J. R. (2004). A dysmorphic boy with 4qter deletion and 4q32.3–34.3 duplication: clinical, cytogenetic, and molecular findings. *American Journal of Medical Genetics*.

